# Dynamic changes in physiological and biochemical properties of flue-cured tobacco of different leaf ages during flue-curing and their effects on yield and quality

**DOI:** 10.1186/s12870-019-2143-x

**Published:** 2019-12-16

**Authors:** Yanjie Chen, Ke Ren, Xian He, Jiangshiqi Gong, Xiaodong Hu, Jiaen Su, Yan Jin, Zhengxiong Zhao, Yanmei Zhu, Congming Zou

**Affiliations:** 1 Yunnan Academy of Tobacco Agricultural Sciences, 33 Yuantong Street, Kunming, Yunnan 650021 People’s Republic of China; 2 College of Tobacco Science, Yunnan Agricultural University, Kunming, 650201 Yunnan China; 3 Faculty of Life Science and Technology, Kunming University of Science and Technology, 727 South Jingming Road, Kunming, 650500 Yunnan China

**Keywords:** Leaf age, Curing process, Tissue structure, Physiological and biochemical change, Economic traits

## Abstract

**Background:**

The leaf age for harvesting flue-cured tobacco leaves is closely related to the quality of tobacco leaves, so an appropriate leaf age for harvesting is important for improving yield and quality of flue-cured tobacco, however, at present, there are few studies on effects of leaf age on physiological and biochemical changes during flue-curing and there is no clear standard of proper leaf ages for harvesting in production.

**Results:**

In the Yunnan tobacco-growing area, an experiment was carried from 2016 to 2017 and different leaf ages were set. The results demonstrate that leaf age has a significant on tissue cell gap, leaf age and flue-curing stages exert significant effects on upper epidermis, palisade and spongy tissue, and leaf thickness of tobacco leaves. The thicknesses of upper and lower epidermis as well as palisade and spongy tissues at different ages show an approximately W-shaped change trend during flue-curing. With the advance of flue-curing stages, contents of starch, chlorophyll, carotenoid, and water in tobacco leaves at different leaf ages decrease, while polyphenol and malondialdehyde (MDA) contents increase. The older the leaf, the faster the chlorophyll, carotenoid, and water contents reduce, while the faster the polyphenol and MDA content rise during flue-curing. The flue-cured tobacco leaves at 116 DAT (days after transplanting) show the highest contents of total nitrogen and nicotine, followed by 123 DAT and those at 130 DAT are the lowest; however, the contents of total sugar and reducing sugar demonstrate a contrary tendency, and the starch content at 116 DAT is much lower than those in the other two treatments. The proportion of superior tobacco, average price, yield, and output value of upper tobacco leaves at different leaf ages are the highest at 123 DAT. The highest sensory evaluation score is found at 123 DAT, while that at 130 DAT is significantly lower in comparison with the other two treatments.

**Conclusions:**

Tobacco leaves harvested at 123 DAT are mature and exhibit a low degree of membrane lipid peroxidation, moderate chemical compositions, and high economic value. 123 DAT improves availability of tobacco leaves.

## Background

Flue-cured tobacco is an important economic crop and timely harvesting and moderate flue-curing are key to ensuring quality. Maturity is regarded as the primary quality factor for grading and is an important index used to measure tobacco quality: tobacco leaf age is one of important factors affecting maturity. Leaf age influences maturity, and further has impacts on flue-curing characteristics of tobacco leaves and yield and quality of flue-cured tobacco leaves. For tobacco, the upper leaves have high economic value but more difficult to get suitable maturity and flue-curing high-quality tobacco because the weather becomes unsuitable for its maturity later in the growing season [1]. Physical properties and chemical and physiological changes in upper tobacco leaves at different ages during flue-curing process have not been studied, so a proper leaf age of upper tobacco leaves has not been determined for harvesting and flue-curing.

Leaf age affects plant growth, and leaf structure and physical properties of plants change accordingly with changes in leaf age. Leaf structure of tobacco leaves is positively correlated with leaf age [2]. Leaves of different ages have differences in morphology and leaf thickness increases on the whole; moreover, the degree of hardening of old leaves is higher than that of young leaves [3]. The leaf structure of tobacco growing in the field increases with the extension of leaf age in a certain range, however, due to double stress of water and nutrient during flue-curing, cells of tobacco leaves dehydrate and shrink during flue-curing and appearance and leaf structure of tobacco leaves change to a significant extent [4]. Research has been conducted into the changes in leaf structure and physical properties of tobacco leaves during flue-curing, while tobacco of different leaf ages during flue-curing is rarely studied.

Leaf age also influences physiological and biochemical changes in plants, thus further affecting plant metabolism. Takuro et al. [5] showed that leaf age affects the photosynthetic rate of tobacco leaves, which further affects photosynthesis of tobacco leaves. The photosynthetic rate of young leaves is higher than that of over-mature old leaves. Some research demonstrates that photosynthetic rate and nitrogen utilisation efficiency show a negative correlation with leaf age, and nitrogen content per unit leaf area decreases with the increasing leaf age [6]. The increase of peroxidase activity is a reliable index for judging maturity and senescence of plants. With increasing leaf age, peroxidase activity in tobacco leaves decreases, while the degree of lignification increases during maturation and senescence [7].

The contents of metabolites in plants vary with leaf age and young leaves are better than old ones in preventing biological and abiotic damage [8]. Hikosaka et al. [9] proposed that the nitrogen content is the highest in newly developed young leaves of grapes and decreases with increasing leaf age. With gradient changes in leaf age, a unique gradient of nitrogen contents can be formed in leaves. In addition, because old leaves are exposed to the environment with CO_2_ for a longer time, non-structural carbohydrate contents of old leaves are higher than those of young leaves [10]. As the leaf ages the contents of chlorophyll and soluble sugar in leaves increase, while the protein content decreases [11]. MDA content represents the aging speed of plants: with the increase of leaf age, the MDA content rises, indicating that the older the leaf, the faster the plants age [12].

Leaf age has certain influences on yield and quality of plants. With the increase of leaf age, the growth time of leaves in the field increases, so the probability of being damaged by climate or insects also rises [13]. Tobacco leaves harvested at different ages exhibit inconsistent maturity. In general, output value, the proportion of superior tobacco, and average price of flue-cured tobacco leaves in upper, middle, and lower parts increase with leaf age. These indices reach the highest when leaves are mature and gradually reduce with increasing age [14]. Furthermore, leaf age also influences flue-curing characteristics of tobacco leaves. Within a certain range, tobacco leaves become easier to flue-cure, while resistance to flue-curing worsens, with the increase of leaf age [15]. Plants harvested too early or late are more susceptible to post-harvest physiological disorders than those harvested at maturity and hence impairing product quality [16].

Physiological and biochemical changes of the upper tobacco leaves at different ages during flue-curing are still unknown and there is no quantitative standard for harvesting mature leaves at a proper age in production. To explore dynamic changes in structure and physiological and biochemical metabolism of upper tobacco leaves at different ages during flue-curing, we collected tobacco samples from seven flue-curing stages from 2016 to 2017. Based on this, metabolic changes to tobacco leaves at different ages during flue-curing and quality of flue-cured tobacco leaves were investigated, so as to propose a standard for harvesting tobacco leaves at a proper leaf age in the tobacco-growing area of Yunnan Province, China.

## Results

### Effects of different leaf ages on morphological characteristics of tobacco leaves

Table 1 shows that leaf age, year and their interaction do not have significant influences on tobacco leaf length and leaf width, but leaf age significantly affects tissue cell gap (*P* < 0.05). The leaf length, leaf width, and tissue cell gap of tobacco leaves increased with the increase of leaf age. Palisade tissue cell gaps at a leaf age of 130 DAT were significantly higher than those at leaf ages of 123 DAT and 116 DAT, while sponge tissue cell gaps in leaf at 116 DAT were significantly lower than in the other two treatments.

**Table 1:** Effects of different leaf ages on morphological characteristics of tobacco leaves

Leaf age (A)	Year (Y)	DF	Leaf length (cm)	Leaf width (cm)	Palisade tissue cell gap (%)	Sponge tissue cell gap (%)
116 DAT	2016		51.2a	16.3a	9.32c	17.25b
123 DAT			54.7a	17.8a	20.34b	31.47a
130 DAT			55.8a	17.5a	29.89a	34.12a
116 DAT	2017		55.6a	18.4a	9.66c	19.60b
123 DAT			61.2a	21.3a	21.81b	34.57a
130 DAT			63.7a	22.6a	28.94a	25.66a
**A**		2	NS	NS	< 0.05	< 0.05
**Y**		1	NS	NS	NS	NS
**A × Y**		2	NS	NS	NS	NS

**Note:** Lowercase letters represent significant differences for different treatments in the same year (*P* < 0.05)

### Effects of different leaf ages on structure of tobacco leaves during flue-curing

#### Effects of different leaf ages on upper epidermis thickness of tobacco leaves during flue-curing

Table 2 shows that leaf age, stage, and interactions of leaf age, year, and stage significantly affect upper epidermis thickness (*P* < 0.05).

**Table 2:** Analysis of variance for the effects of the age of tobacco leaves, year, stage and their interactions on tissue structure

Effect/contrast	DF	Upper epidermis	Lower epidermis	Palisade tissue	Spongy tissue	Leaf thickness
		-------------Probability of a greater *F-*value--------------				
Leaf age (L)	2	0.0049	0.4107	<.0001	0.0025	0.0003
Year (Y)	1	0.0003	0.0411	0.2758	0.0533	0.1895
Stage (S)	6	<.0001	<.0001	<.0001	<.0001	<.0001
L*Y	2	0.093	0.2566	0.5202	0.1901	0.1859
L*S	12	0.0035	0.6867	0.0077	0.4023	0.0938
Y*S	6	0.0796	0.015	0.6096	0.3335	0.2378
L*Y*S	12	0.0383	0.894	0.4387	0.3115	0.273

**Fig. 1:**
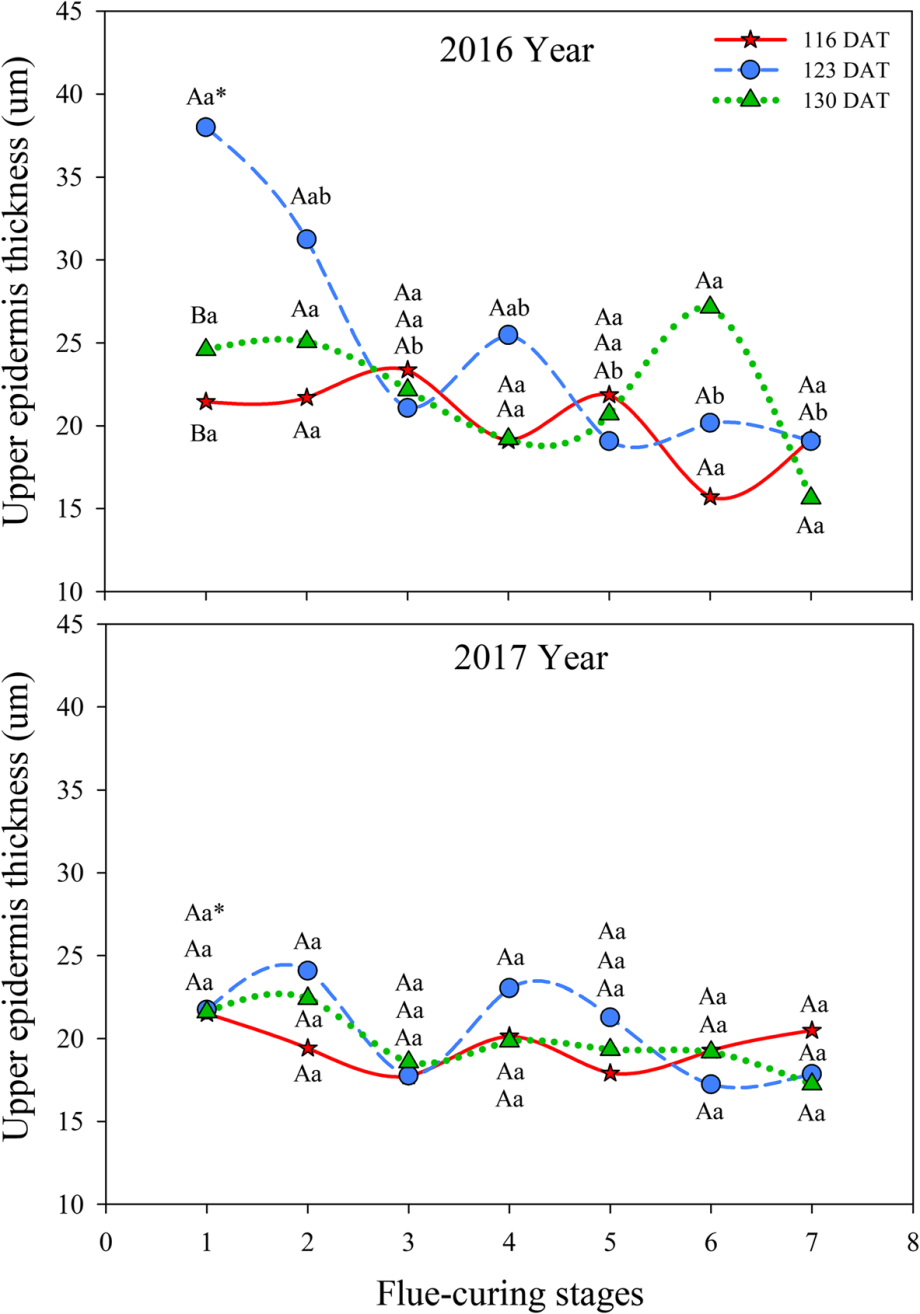
Effects of interactions of different leaf ages, flue-curing stages, and years on upper epidermis thickness. Note: different capital letters indicate the significant differences between different treatments at the same stage. Different lowercase letters denote the significant differences between different stages of the same treatment. The asterisk indicates the significant differences between different years under the same treatments and same period (same below)

As shown in Fig. 1, the upper epidermis thicknesses of tobacco leaves at different ages gradually decreased during flue-curing and approximately showed a tendency to first decrease, then slightly increase, and finally decrease. In 2016, the upper epidermis thickness in 123 DAT rapidly reduced in Stages 1 to 3. The upper epidermis thickness in Stage 3 was significantly lower than that in Stage 1, while that in Stage 4 rose slightly then slowly decreased. At 130 DAT, the upper epidermis thickness gradually decreased in Stages 1 to 4, while it rose slightly in Stages 5 and 6 and then rapidly reduced after Stage 6. The upper epidermis thickness at 116 DAT rapidly decreased in Stages 3 to 6, while it rose slightly in Stages 6 and 7. In 2017, the upper epidermis thicknesses in Stages 1 to 3 in the three treatments showed a decreasing trend, and the thickness reduced slightly by 116 DAT. The thickness rapidly decreased and then slightly rose in Stages 4 to 6 at 123 DAT. Moreover, the thickness slowly decreased after Stage 4 in 116 DAT and 130 DAT.

#### Effects of different leaf ages on lower epidermis thickness of tobacco leaves during flue-curing

Table 2 demonstrates that year and flue-curing stage as well as their interactions significantly influence lower epidermis thickness of tobacco leaves (*P* < 0.05).

**Fig. 2:**
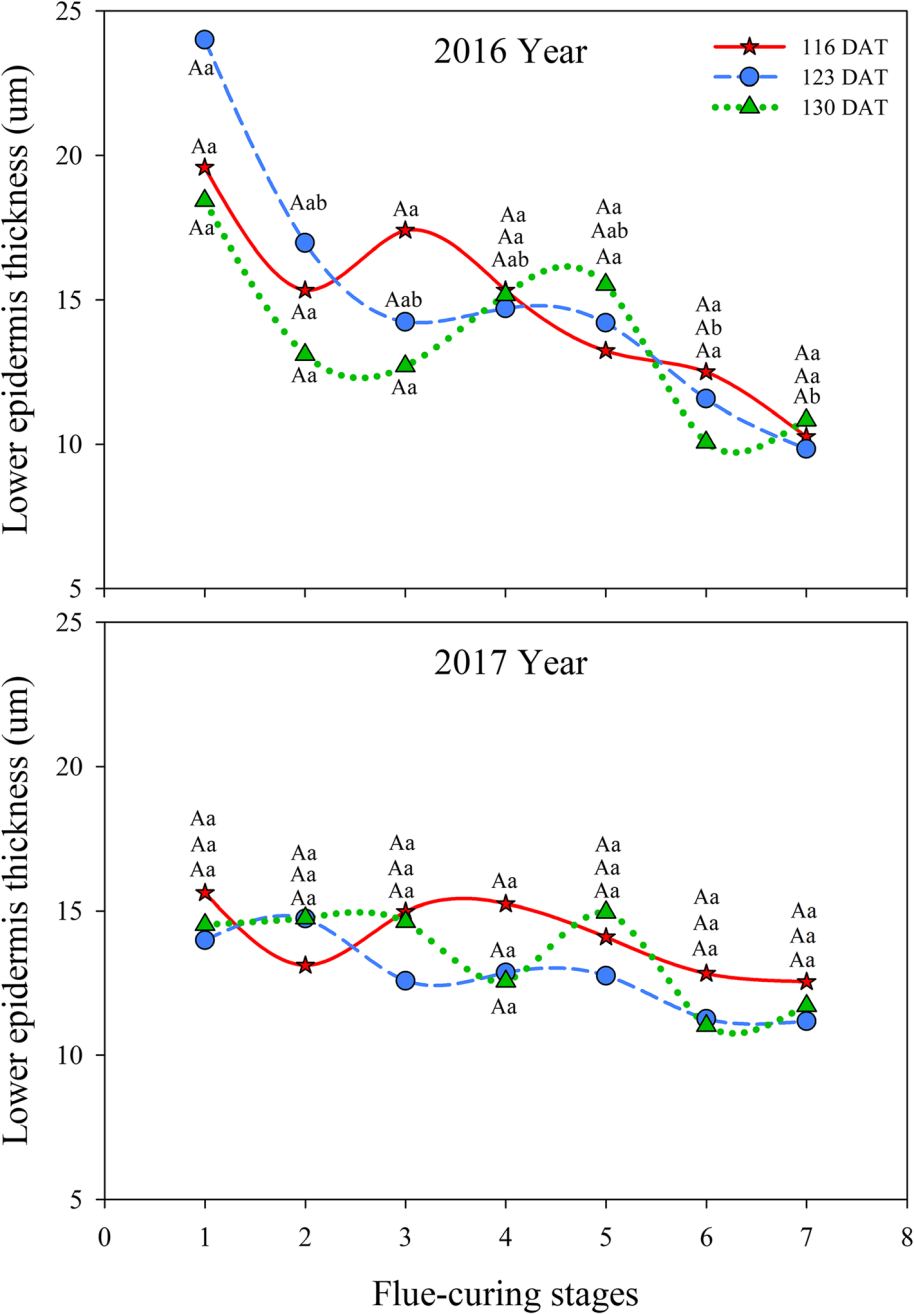
Effects of interactions of different leaf ages, flue-curing stages, and years on lower epidermis thickness

It can be seen from Fig. 2 that lower epidermis thickness gradually reduced with the advance of flue-curing. Within two years, the lower epidermis thickness at 123 DAT and 130 DAT rapidly reduced in Stages 1 to 4, while it rose slightly in Stages 4 and 5, then slowly decreased thereafter Stage 5. The decreases in Stages 1 to 4 at 123 DAT and 130 DAT in 2016 were greater than in 2017. The lower epidermis thickness at 116 DAT rose slightly before Stage 3 and then rapidly decreased in Stages 3 to 7.

#### Effects of different leaf ages on palisade tissue thickness of tobacco leaves during flue-curing

As displayed in Table 2, leaf age and flue-curing stage exert significant effects on palisade tissue thickness (*P* < 0.05).

**Fig. 3:**
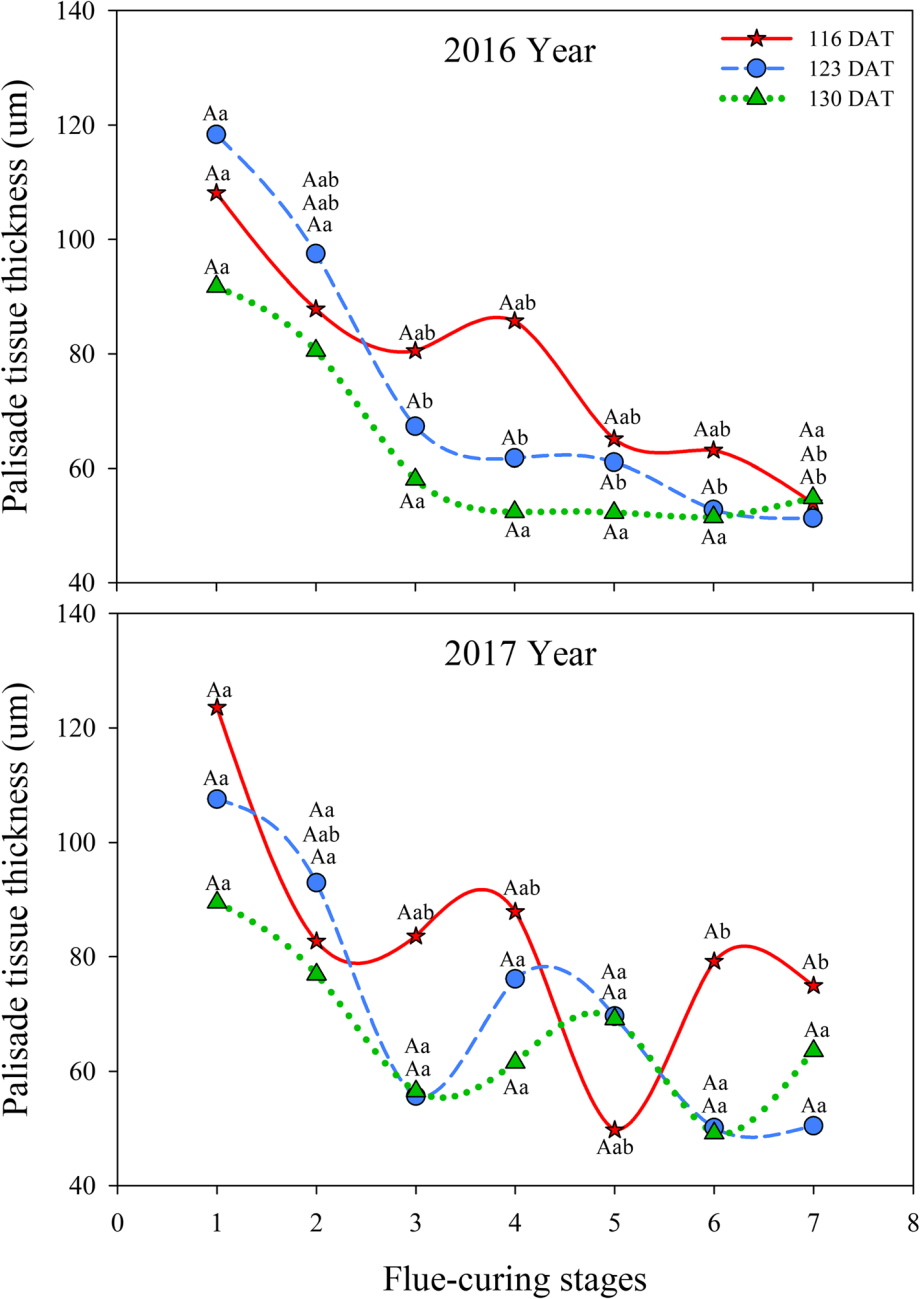
Effects of interactions of different leaf ages, flue-curing stages, and years on palisade tissue thickness of tobacco leaves

As demonstrated in Fig. 3, palisade tissue thicknesses at 123 DAT and 130 DAT decreased with flue-curing in 2016, of which the thickness rapidly decreased in Stages 1 to 3 and then more slowly after Stage 4. At 116 DAT, the thickness rapidly decreased in Stages 1 to 3, then rose slightly, then slowly reduced in Stages 3 and 4. In 2017, the palisade tissue thickness in different treatments first decreased, then increased and finally decreased in flue-curing stages: however, the thicknesses at 123 DAT and 130 DAT fell quickly in Stages 1 to 3, while slowly decreasing after a slight increase in Stages 3 to 5, then slowly rising again in Stages 6 and 7. At 116 DAT, the thickness quickly decreased in Stages 1 and 2 and 4 and 5, then slightly increased in Stages 2 to 4 and 5 and 6.

#### Effects of different leaf ages on spongy tissue thickness of tobacco leaves during flue-curing

Table 2 shows that leaf age and flue-curing stage as well as their interaction show significant effects on spongy tissue thickness (*P* < 0.05).

**Fig. 4:**
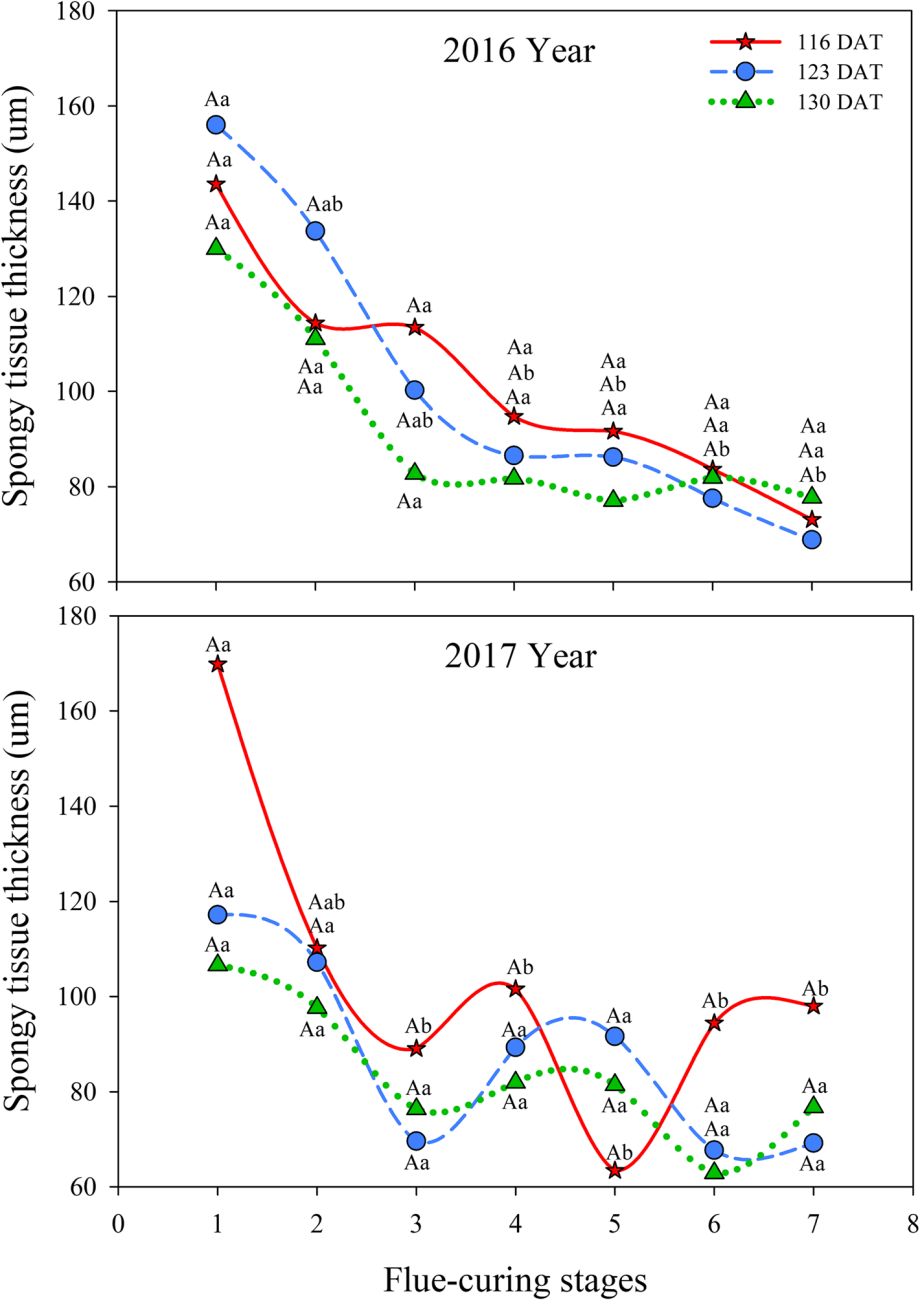
Effects of interactions of different leaf ages, flue-curing stages, and years on spongy tissue thickness of tobacco leaves

It can be observed from Fig. 4 that the spongy tissue thicknesses in the three treatments were all reduced by flue-curing in 2016. Spongy tissue thicknesses at 123 DAT and 130 DAT rapidly decreased in Stages 1 to 3 and then slowly reduced, while the decrease at 116 DAT during flue-curing was smaller than those in the other two treatments. In 2017, the spongy tissue thicknesses in the three treatments rapidly declined in Stages 1 to 3. The thicknesses of spongy tissue in treatments at 123 DAT and 130 DAT rose slightly, then slowly decreased in Stages 3 to 5, while that at 116 DAT were rapidly reduced after undergoing a slight increase in Stages 3 and 4, and then slowly increased again in Stages 5 and 6.

#### Effects of different leaf ages on tobacco leaf thickness during flue-curing

Table 2 displays that leaf age and flue-curing stage significantly influence leaf thickness (*P* < 0.05).

**Fig. 5:**
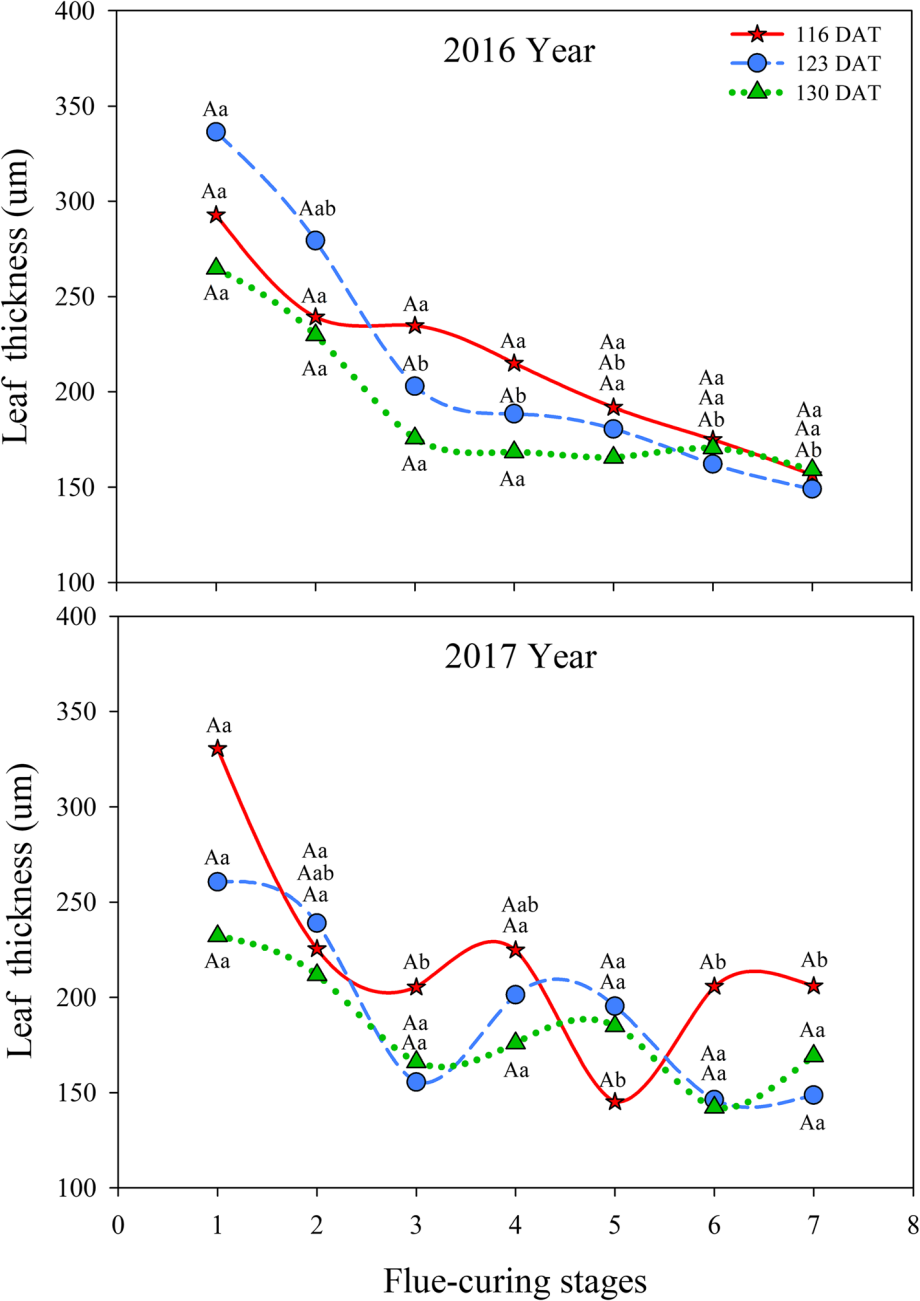
Effects of interactions of different leaf ages, flue-curing stages, and years on tobacco leaf thickness

As displayed in Fig. 5, during the two years, leaf thicknesses in the three treatments all rapidly decreased in flue-curing Stages 1 to 3, while the reduction tended to be smaller in other stages. In 2016, leaf thickness in treatments of different leaf ages showed a stable decrease during flue-curing. In 2017, the leaf thickness in treatments of different leaf ages firstly reduced and then slightly rose and decreased during flue-curing.

### Effects of different leaf ages on physiological and biochemical indices of tobacco leaves during flue-curing

#### Effects of different leaf ages on starch contents in tobacco leaves during flue-curing

Table 3 demonstrates that leaf age, flue-curing stage, and year as well as their interactions have significant effects on the starch content (*P* < 0.05).

**Table 3:** Analysis of variance for the effects of the age of tobacco leaves, year, stage and their interactions on physiological changes

Effect/contrast	DF	Starch	Chlorophyll a	Chlorophyll b	Carotenoid	Water	Polyphenol	MDA
		-------------------------Probability of a greater *F*-value------------------------						
Leaf age (L)	2	<.0001	<.0001	<.0001	<.0001	<.0001	<.0001	<.0001
Year (Y)	1	<.0001	<.0001	<.0001	<.0001	0.0202	<.0001	<.0001
Stage (S)	6	<.0001	<.0001	<.0001	<.0001	<.0001	<.0001	<.0001
L*Y	2	<.0001	<.0001	<.0001	<.0001	0.0674	<.0001	<.0001
L*S	12	<.0001	<.0001	<.0001	<.0001	<.0001	<.0001	<.0001
Y*S	6	<.0001	<.0001	<.0001	<.0001	0.0045	<.0001	<.0001
L*Y*S	12	<.0001	<.0001	<.0001	<.0001	0.0023	<.0001	<.0001

**Fig. 6:**
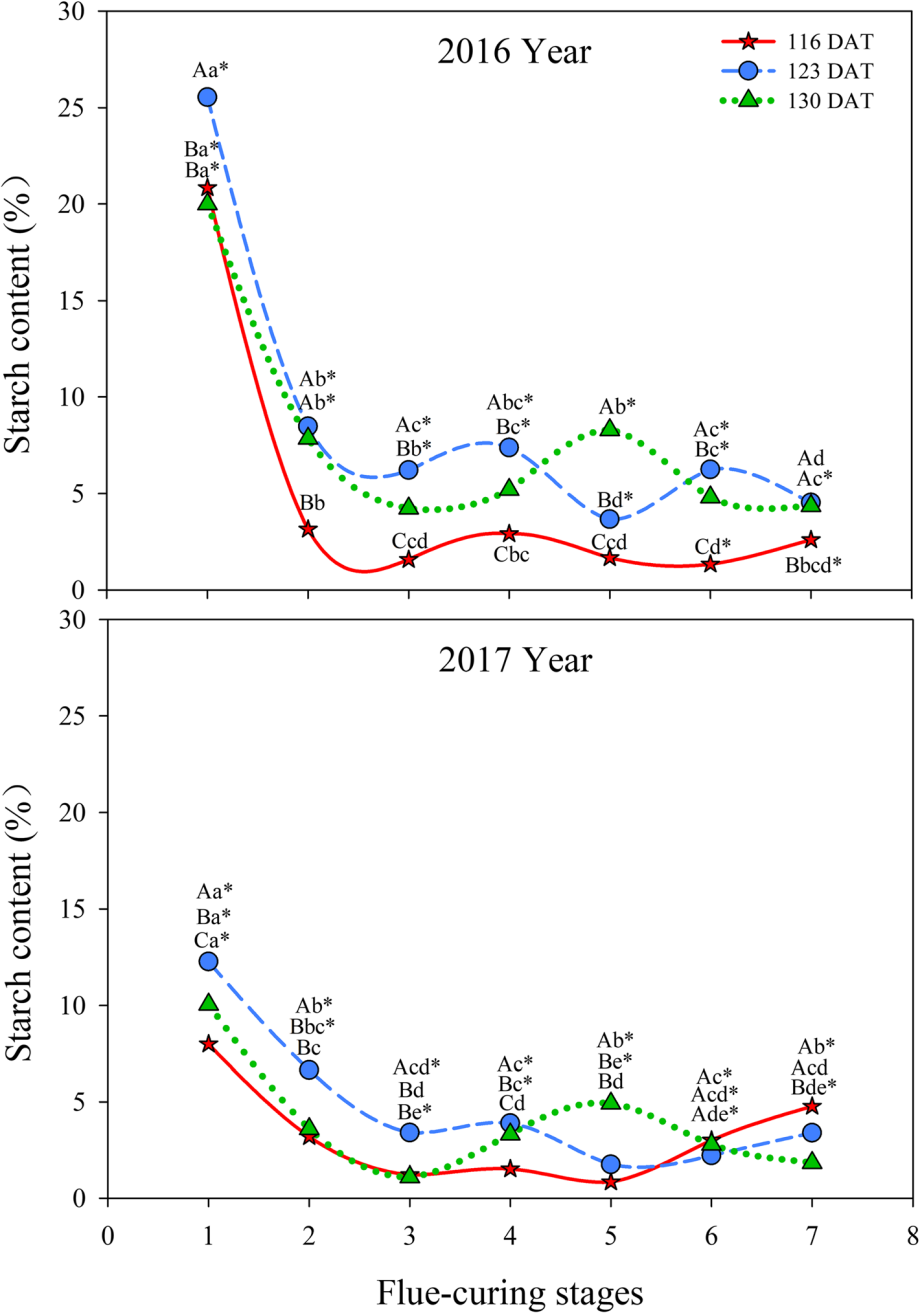
Effects of interactions of different leaf ages, flue-curing stages, and years on starch contents of tobacco leaves

As shown in Fig. 6, the 123 DAT group showed the highest starch content in flue-curing Stage 1, followed by 116 DAT and 130 DAT during the two years. In Stages 1 to 3, starch contents rapidly decreased in different treatments and those of the three treatments in Stage 3 were significantly lower in comparison with Stage 1. Starch contents at 116 DAT and 123 DAT rapidly reduced again in Stages 4 to 5, while those at 130 DAT rose slightly, then slowly decreased in these stages.

#### Effects of different leaf ages on chlorophyll contents during flue-curing

Table 3 indicates that leaf age, flue-curing stage and year as well as their interactions exert significant effects on contents of chlorophylls a and b (*P* < 0.05).

**Fig. 7:**
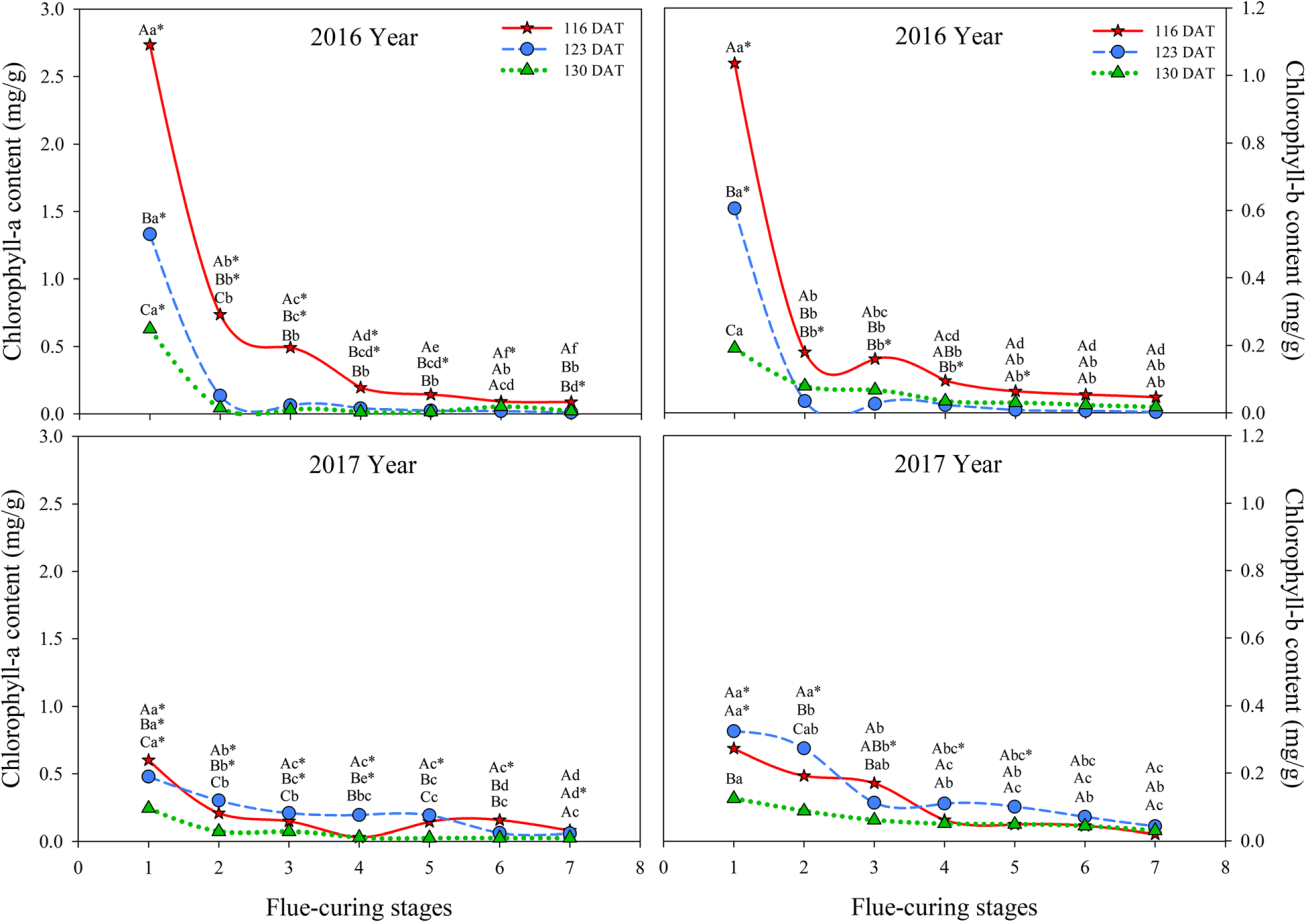
Effects of interactions of different leaf ages, flue-curing stages, and years on chlorophyll contents of tobacco leaves

It can be seen from Fig. 7 that contents of chlorophylls a and b in different treatments decreased during flue-curing. Contents of chlorophyll a in different treatments showed significant differences in flue-curing Stages 1 and 2 and the decrease in chlorophyll a content in Stages 1 and 2 of the three treatments in 2016 was larger than that in 2017. Contents of chlorophyll a slowly decreased in other stages after Stage 2. Contents of chlorophyll b in different treatments largely reduced in Stages 1 and 2 and the decrease in 2016 was greater than that in 2017. Contents of chlorophylls a and b at 116 DAT in each stage in 2016 were higher than those in the other two treatments and the contents thereof in Stages 3 and 4 at 116 DAT were higher than those in the other treatments.

#### Effects of different leaf ages on carotenoid contents during flue-curing

According to Table 3, leaf age, flue-curing stage, and year as well as their interactions have significant impacts on carotenoid contents (*P* < 0.05).

**Fig. 8:**
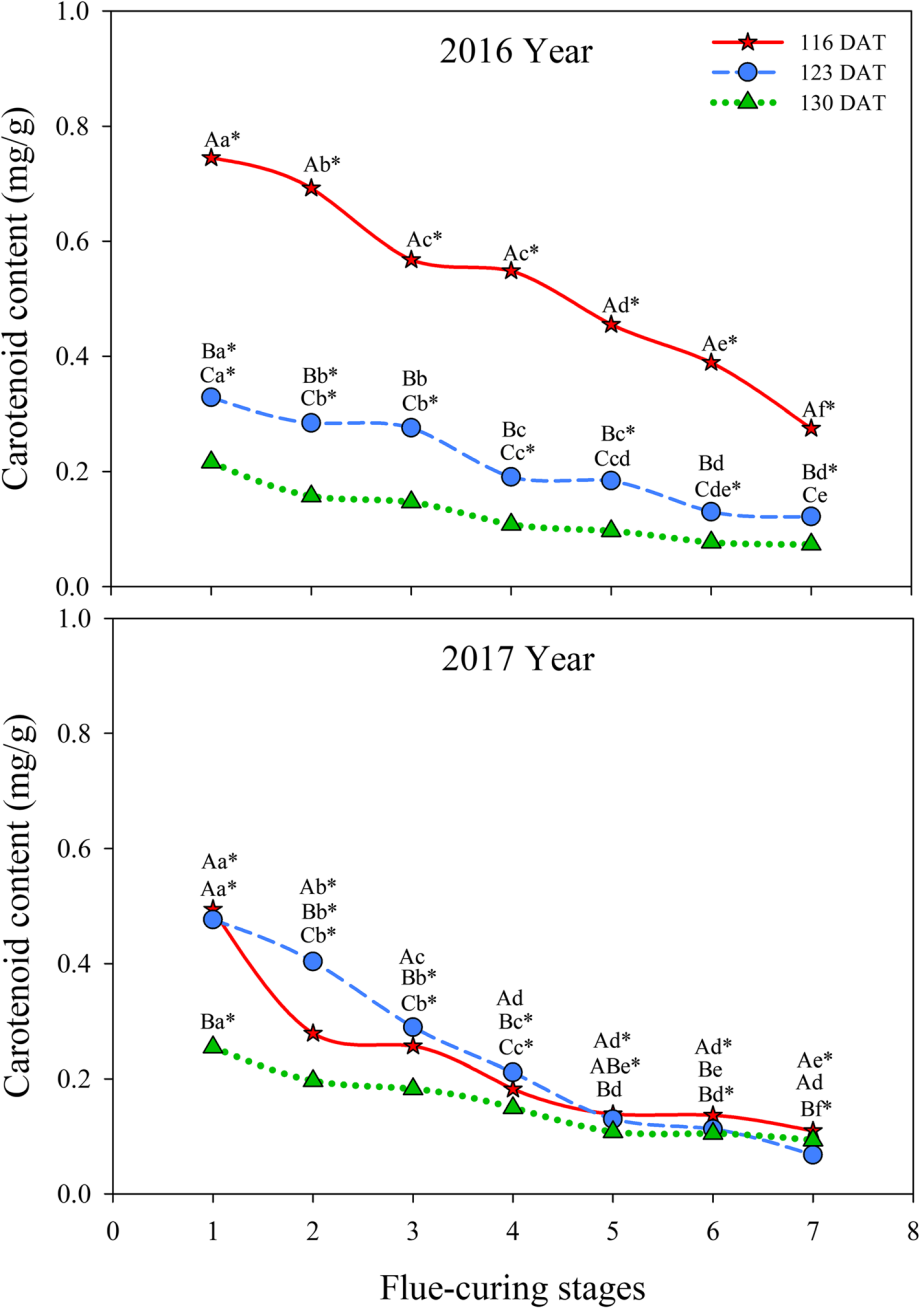
Effects of interactions of different leaf ages, flue-curing stages, and years on carotenoid contents of tobacco leaves

As shown in Fig. 8, carotenoid contents in different treatments decreased during flue-curing. Carotenoid contents at 116 DAT were greater than those in the other two treatments in each flue-curing stage in 2016. The three treatments were such that the carotenoid contents largely decreased in flue-curing Stages 1 to 3 in 2016 and 2017, of which the content in Stage 3 was significantly lower than those in Stage 1 and the rate of change decreased in the three treatments after Stage 3. After flue-curing, the three treatments demonstrated that carotenoid contents in Stage 7 were significantly different and the highest content was found at 116 DAT during the two years.

#### Effects of different leaf ages on water contents during flue-curing

In accordance with Table 3, the interaction between leaf age and flue-curing stage as well as between year and flue-curing stage significantly affect water contents (*P* < 0.05).

**Fig. 9:**
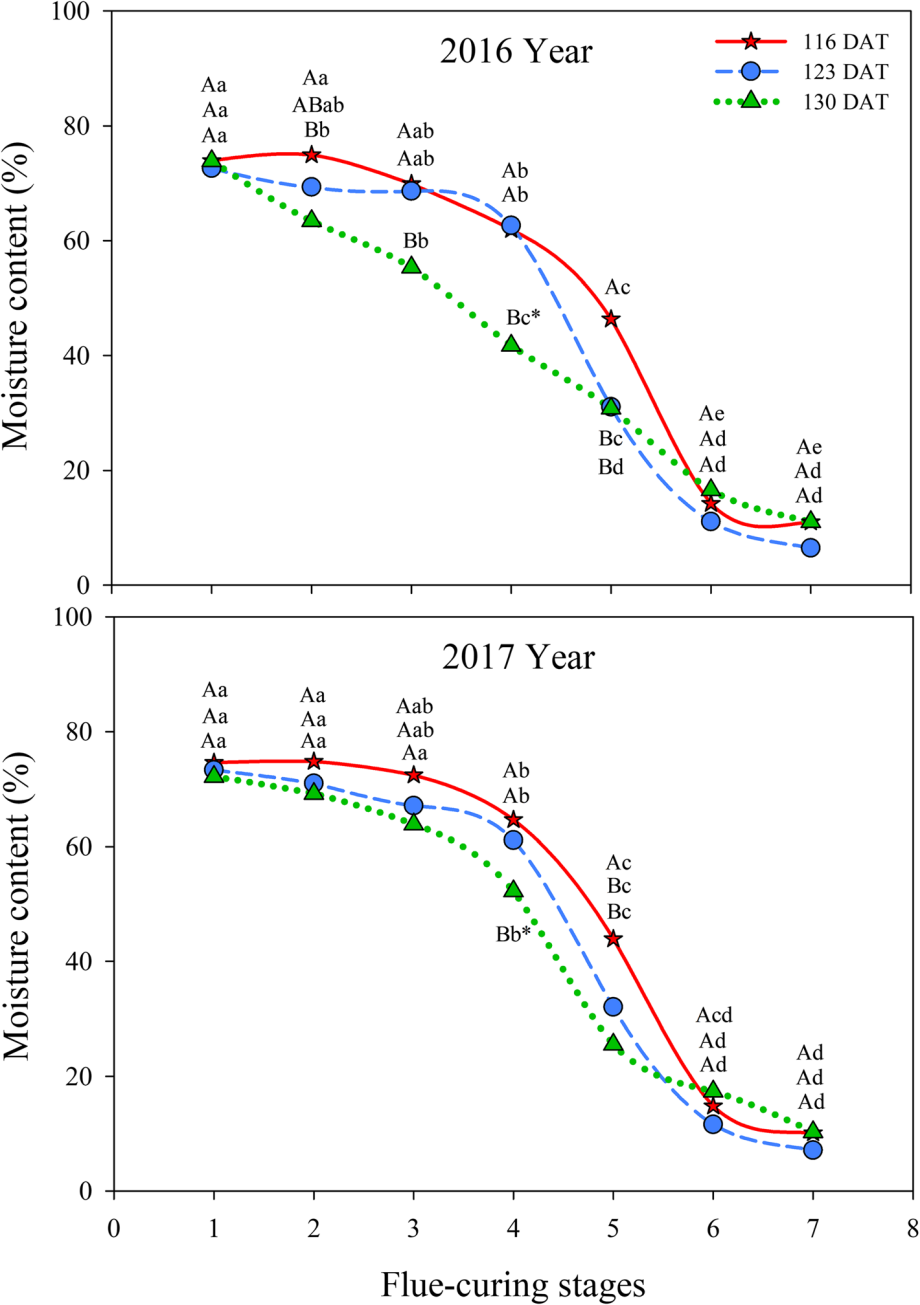
Effects of interactions of different leaf ages, flue-curing stages, and years on moisture contents of tobacco leaves

As shown in Fig. 9, with the advance of flue-curing, the moisture content in tobacco leaves in treatments of different ages gradually reduced. Moisture was lost slowly at first, then fast, and then slowly in tobacco leaves in different treatments during flue-curing. Less moisture in tobacco leaves was lost at a slower rate in Stages 1 to 4, while tobacco leaves lost a lot of moisture and showed a rapid rate of moisture loss in Stages 4 to 6. Moreover, the moisture loss rate of tobacco leaves slowed down in Stages 6 and 7. In different treatments, moisture contents in Stage 6 were lower than those in Stages 1 to 5. In Stages 4 to 6, moisture was lost fastest at 123 DAT, followed by 116 DAT, and 130 DAT.

#### Effects of different leaf ages on polyphenol contents during flue-curing

Table 3 shows that leaf age, flue-curing stage, and year as well as their interactions significantly influence polyphenol contents (*P* < 0.05).

**Fig. 10:**
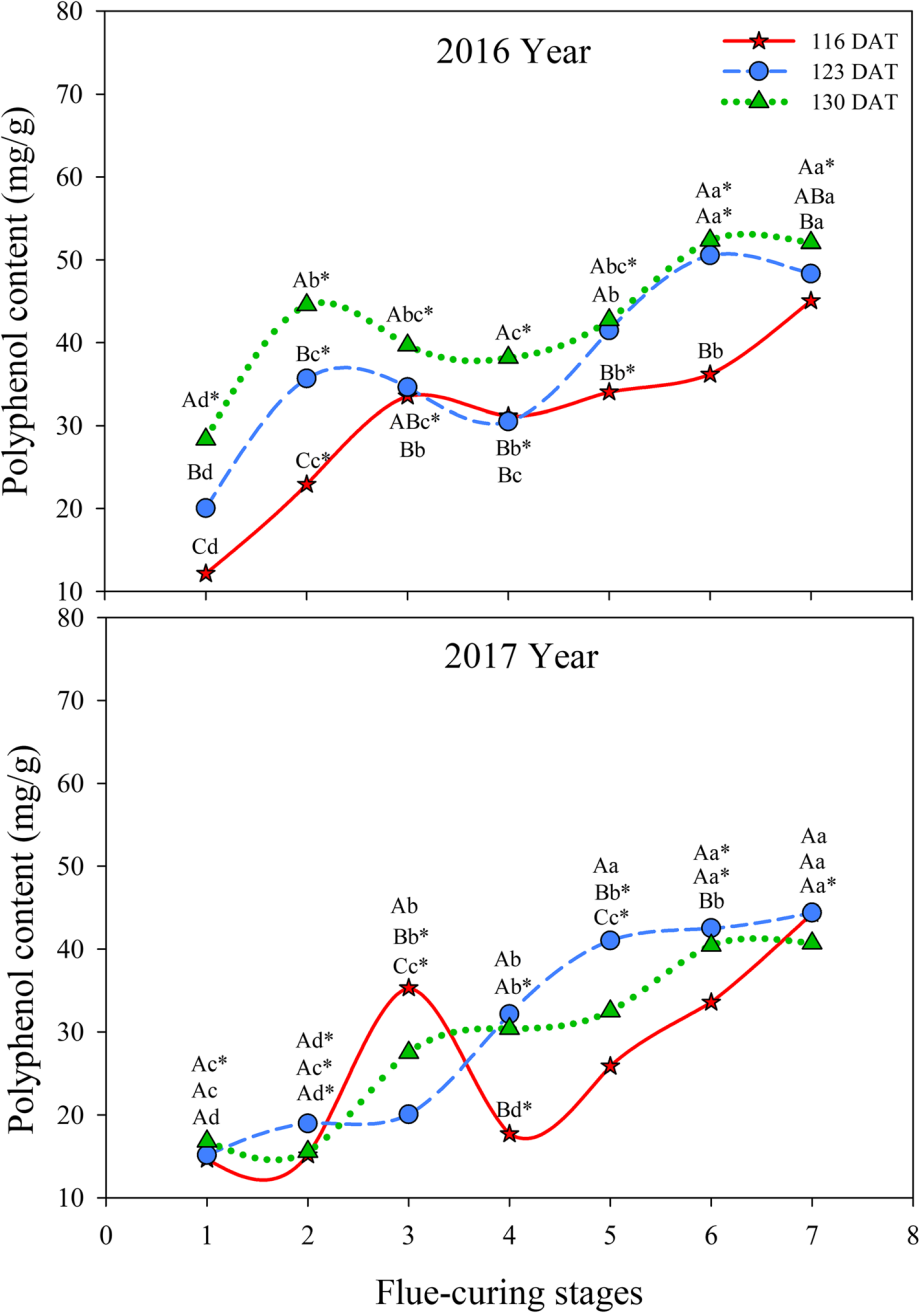
Effects of interactions of different leaf ages, flue-curing stages, and years on polyphenol contents of tobacco leaves

As demonstrated in Fig. 10, polyphenol contents in different treatments gradually rose during flue-curing and the contents thereof at 123 DAT and 130 DAT were higher than that at 116 DAT on the whole. In 2016, polyphenol contents in different treatments significantly increased in Stages 1 to 2 and rose slowly rose after decreasing in Stages 3 and 4. Polyphenol contents in Stage 7 were significantly higher than those in other stages. In 2017, polyphenol contents in Stages 3 and 4 rapidly increased at 123 DAT and 130 DAT and were significantly higher than those in Stages 1 and 2. Moreover, the content thereof at 116 DAT rapidly increased in Stages 1 to 3 and then rose gradually after a large decrease in Stages 3 and 4.

#### Effects of different leaf ages on MDA contents during flue-curing

Based on Table 3, leaf age, flue-curing stage, and year as well as their interactions exert significant influences on MDA content (*P* < 0.05).

**Fig. 11:**
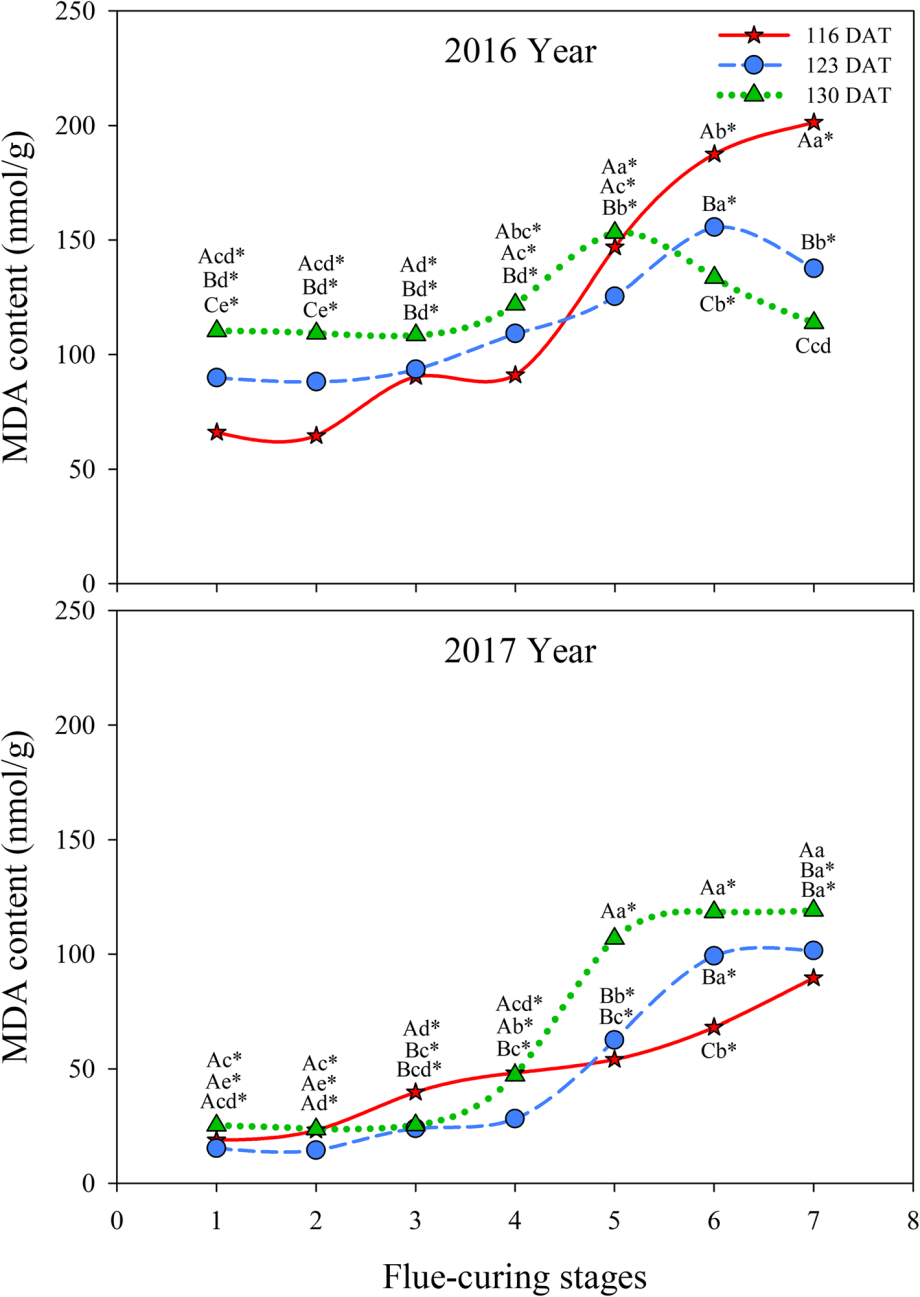
Effects of interactions of different leaf ages, flue-curing stages, and years on MDA contents of tobacco leaves

It can be seen from Fig. 11 that MDA contents in different treatments gradually increased during flue-curing. MDA contents in different treatments rapidly rose in Stages 3 to 5 and then the increase slowed down thereafter. The rising MDA content at 116 DAT was greater than those in the other two treatments in 2016, while the amplitude at 130 DAT was larger than those in the other treatments in 2017.

### Effects of different leaf ages on interior quality of flue-cured tobacco leaves

#### Effects of different leaf ages on total sugar and reducing sugar in flue-cured tobacco leaves

In accordance with Table 4, leaf age and year as well as their interaction have significant effects on the contents of total sugar (*P* < 0.05), and leaf age and its interaction with year significantly affect the contents of reducing sugar (*P* < 0.05).

**Table 4:** Effects of the leaf age, year, and their interactions on chemical compositions and sensory evaluation score

Leaf age (A)	Year (Y)	DF	Total sugar content (%)	Reducing sugar content (%)	Nitrogen content (%)	Nicotine content (%)	Starch content (%)	Polyphenol content (mg/g)	Sensory evaluation score
123 DAT			27.33 a	24.41 a	2.46 b	3.57 a*	6.3 a*	26.21 a	90.5 a
130 DAT	2016		28.19 a	26.08 a*	2.05 b	3.09 b*	6.62 a*	19.81 b*	85 b
116 DAT	2017		29.56 a*	22.21 a*	2.32 a*	2.19 a*	2.65 b*	29.87 a*	90 a
123 DAT			26.19 a	22.45 a	2.18 a	2.38 a*	4.43 a*	31.98 a	91 a
130 DAT			29.73 a	20.03 a*	2.26 a	2.32 a*	2.81 b*	28.94 a*	85 b
A		2	**	**	**	**	**	**	**
Y		1	**	NS	**	**	**	**	NS
A × Y		2	**	**	**	**	**	NS	NS

**Note:** Lowercase letters represent that differences are significant in different treatments in the same year (*P* < 0.05); * indicates the significant difference in the same treatment in different years (*P* < 0.05); ** denotes the significant differences of each factor and their interactions on this index

In 2016, the contents of total sugar and reducing sugar at 116 DAT were significantly lower than those in the other two treatments. In 2017, the contents of total sugar were the highest at 130 DAT, followed by 116 DAT, while the lowest contents were found at 123 DAT. Moreover, 123 DAT, 116 DAT, and 130 DAT were ranked from high to low according to the contents of reducing sugar. Under the same treatment, contents of total sugar and reducing sugar at 116 DAT were significantly different in different years and the contents of reducing sugar at 130 DAT showed significant differences in different years.

#### Effects of different leaf ages on total nitrogen and nicotine in flue-cured tobacco leaves

Table 4 shows that leaf age, year and their interaction significantly influence contents of total nitrogen and nicotine (*P* < 0.05). In 2016, contents of total nitrogen at 116 DAT were significantly higher than those in the other two treatments, while nicotine contents at 130 DAT were significantly lower than those elsewhere. In 2017, the contents of total nitrogen were the highest at 116 DAT, followed by that at 130 DAT and the lowest contents were found at 123 DAT: however, nicotine contents peaked at 123 DAT, followed by 130 DAT and 116 DAT. Under the same treatment, the contents of total nitrogen at 116 DAT demonstrated significant differences in different years and nicotine contents in the three treatments were significantly different in different years.

#### Effects of different leaf ages on starch contents in flue-cured tobacco leaves

Table 4 shows that leaf age, year, and their interaction significantly influence starch contents (*P* < 0.05). In 2016, starch contents at 116 DAT were significantly lower than those in the other treatments, while the contents at 123 DAT were much higher than those in the other two treatments in 2017. Under the same treatment, starch contents in different years were significantly different.

#### Effects of different leaf ages on polyphenol contents in tobacco leaves

Table 4 demonstrates that leaf age and year have significant influences on polyphenol contents (*P* < 0.05). In 2016, polyphenol contents at 123 DAT were significantly higher than those elsewhere, while differences in polyphenol contents in different treatments were not significant in 2017. The polyphenol contents were the highest at 116 DAT, followed by that at 123 DAT and the lowest contents were found at 130 DAT. Under the same treatment, polyphenol contents at 116 DAT and 130 DAT were significantly different in different years.

#### Effects of different leaf ages on sensory evaluation scores of flue-cured tobacco leaves

It can be observed from Table 4 that leaf age affects sensory evaluation scores of tobacco leaves (*P* < 0.05). Sensory evaluation scores at 130 DAT were significantly lower than those in the other two treatments in 2016 and 2017. In both years, sensory evaluation scores were the highest at 123 DAT, followed by 116 DAT, while 130 DAT had the lowest scores. Same treatment exhibited insignificant differences of sensory evaluation scores in different years. Sensory evaluation scores at 116 DAT and 123 DAT in 2017 were higher than those in 2016, while the score at 130 DAT in 2016 was higher than that in 2017.

#### Effects of different leaf ages on economic traits of flue-cured tobacco leaves

#### Effects of different leaf ages on yield of upper tobacco leaves

As shown in Table 5, year has significant effects on yield of upper tobacco leaves (*P* < 0.05). In the same year, yield differences were insignificant in different treatments and the highest yield was found at 123 DAT in 2016 and 2017. Under the same treatment, yields at 123 DAT and 130 DAT were significantly different in different years and yields in the three treatments in 2016 were higher than those in 2017.

**Table 5:** Effects of the leaf age, year, and their interactions on economic traits of upper leaves

Leaf age (A)	Year (Y)	DF	Yield (kg/ha)	Average price (dollar/kg)	Output values (dollar/ha)	Proportions of superior tobacco (%)
123 DAT			860.56 a*	3.54 a	3036.63 a	66.42 a
130 DAT	2016		850.67 a*	3.5 a	2981.07 a	65.58 a
116 DAT	2017		743.3 a	3.53 a	2631.96 a	62.68 a
123 DAT			754.9 a*	3.63 a	2741.61 a	65.64 a
130 DAT			728.19 a*	3.47 a	2533.44 a	62.34 a
A		2	NS	NS	NS	NS
Y		1	**	NS	NS	NS
A × Y		2	NS	NS	NS	NS

**Note:** Lowercase letters represent significant differences for different treatments in the same year (*P* < 0.05); * indicates significant differences for the same treatment in different years (*P* < 0.05); ** denotes the significant difference of each factor and their interaction on the index

#### Effects of different ages on average price of upper tobacco leaves

Table 5 demonstrates that average prices in different treatments in the same year differed insignificantly and they were the highest at 123 DAT in 2016 and 2017. Significant differences in average prices were not found in the same treatment in different years. In 2017, average prices at 116 DAT and 123 DAT were higher than those in 2016 and 130 DAT showed the highest average prices in 2016.

#### Effects of different leaf ages on output value of upper tobacco leaves

As displayed in Table 5, leaf age and year, as well as their interaction did not significantly affect the output value of upper tobacco leaves. The highest output value was found at 123 DAT in 2016 and 2017. Output values at 123 DAT in 2016 were 1.86 and 6.28% higher than those at 130 DAT and 116 DAT and 8.22 and 4.17% higher than those at 130 DAT and 116 DAT in 2017. Output values in different years in the same treatment showed insignificant differences and the three treatments showed that output value in 2016 was higher than that in 2017.

#### Effects of different leaf ages on the proportion of superior tobacco of upper tobacco leaves

It can be seen from Table 5 that leaf age, year, and their interaction have no significant influence on the proportion of superior tobacco. In the same year, the proportions of superior tobacco showed insignificant differences in different treatments and the highest proportions of superior tobacco in 2016 and 2017 were found at 123 DAT. Under the same treatment, the differences in the proportions of superior tobacco in different years were insignificant, while the proportions in the three treatments in 2016 were higher than those in 2017.

## Discussion

### Morphological characteristics of tobacco leaves at different leaf age

Different leaf age tobacco leaves have different morphological characteristics. For example, the leaf length, and leaf width of old tobacco leaves exceed those of young tobacco leaves, at present, farmers, in tobacco leaf production, judge whether the tobacco leaves are mature or not mainly according to tobacco blade colour, main vein colour and villus condition. In a certain growing period, the length and width of tobacco leaves are variable and rainfall, temperature and soil condition will affect the growth of leaf length and width [17]. In this research, leaf length and leaf width did not show any significant difference between different leaf age, because tobacco leaf length and width did not increase continuously with growing time [18]. Therefore, tobacco leaf length and width can be regarded as reference indicators for tobacco maturity but not judgement indicators.

Tissue cell gap is an indirect reflection of tobacco leaf age as it increases with the senescence of tobacco [19]. The older the leaf, the bigger the tissue cell gap. In this research, for palisade tissue cell gaps, different leaf age tobacco leaves had a significant difference, 130 DAT leaf age treatments have the maximum value of palisade tissue cell gap in 2016 and 2017. For sponge tissue cell gap, a leaf age of 116 DAT was significantly lower than other two leaf age treatments, and a leaf age of 123 DAT and 130 DAT did not make a significant difference because, when the tobacco leaf is younger, their cell tissue structure is tight, and the tissue cell gap is small. While when the tobacco leaf ages, the cells shrink, and the tissue cell gap decreases [20].

### Structural changes in tobacco leaves at different ages during flue-curing

The structure of tobacco leaves firstly depends on leaf age for harvesting fresh tobacco leaves, because the age of fresh tobacco leaves determines maturity, while maturity sets the framework of the structure of tobacco leaves. Moreover, structure is limited by flue-curing [20, 21]. Changes of leaf thickness and structure are mainly related to dehydration and inclusion changes. During flue-curing, dehydration of tobacco leaves and inclusion changes mainly occur in the yellowing stage and leaf-drying stage [22]. In this experiment, reduction of leaf thickness is mainly shown in Stages 2 to 5 during flue-curing, that is, the yellowing, and early leaf-dying stages. The lower epidermis thickness at 116 DAT increases before Stage 3. Some studies [23] also show that the lower epidermis thickness of immature tobacco leaves increases before rising to 38 °C during flue-curing. Based on Figs. 1 to 6, the thinning amplitudes of palisade and spongy tissues are much greater than those of the upper and lower epidermis during flue-curing, indicating that changes in leaf thickness mainly result from palisade and spongy tissues. During flue-curing, palisade and spongy tissues in the three treatments rapidly shrink in Stages 1 to 3 because peak shrinkage of palisade and spongy tissues during flue-curing is found between 35 and 38 °C, during which the acceleration of drying of tobacco leaves results in rapid shrinkage of palisade and spongy tissues [24]. During flue-curing, leaf thickness slightly increases in Stages 6 and 7, because leaf thickness slightly increases after tobacco leaves regain moisture and absorb water. Leaf thickness is a sensitive to water conditions of plants. When leaves lose water and shrink, leaf thickness reduces, while it moderately rises when leaves absorb water and expand [25].

### Physiological and biochemical changes of tobacco leaves at different ages during flue-curing

Plastid pigments mainly include chlorophyll and carotenoid, which are important aroma precursors influencing appearance and quality of tobacco leaves. In flue-curing Stage 1, chlorophyll and carotenoid contents are the highest at 116 DAT, followed by 123 DAT, while the lowest contents are found at 130 DAT, because pigment contents reduce during the maturation process [26]. Degradation rates of chlorophylls at 116 DAT during flue curing are faster than those at 123 DAT and 130 DAT, which results from the too-young leaf age, so that chlorophyll degradation in tobacco leaves with a low maturity is higher than that of tobacco leaves with a high maturity [27]. It can be found (Figs. 7 and 8) that chlorophyll and carotenoid contents are significantly different in different years. The reason is that degradation and transformation of plastid pigments are greatly affected by climatic conditions, such as temperature, light, and rainfall [28], while the region showed different climatic conditions in 2016 and 2017. In different treatments, a lot of water is lost quickly in Stages 4 to 6, which is the late yellowing stage and leaf-drying stage during flue-curing. During this period, a lot of chlorophylls in tobacco leaves degrade, dehydrate and dry, and decrease rate of water in this period is fast [29].

The senescence of plant tissue is always accompanied by the damage to intracellular membrane structures and the MDA content indicates the extent of damage to the membrane. In flue-curing Stages 3 to 6, MDA contents of tobacco leaves at different ages increase greatly, while water contents of tobacco leaves decrease in the same period. Some research shows that the changes in MDA contents during flue-curing are closely correlated with dehydration rate. Rapid dehydration of tobacco leaves is the direct cause of drastic membrane lipid peroxidation and increase of MDA contents [30]. In the whole flue-curing stage in 2016, the polyphenol content was the highest at 130 DAT, followed by that at 123 DAT, while that at 116 DAT showed the lowest content; however, 123 DAT, 130 DAT, and 116 DAT were ranked from high to low according to polyphenol content in 2017. This is because more mature the tobacco leaf in the field, the higher the polyphenol content; however, when the tobacco leaves are completely mature, polyphenol contents reduce and are affected by environmental factors [31]. During flue-curing, due to thermolysis of phenolic glycoside and enzymatic decomposition, phenolic substances of flue-cured tobacco change to significant extents; because of pyrolytic conversion of pyrolysates, such as lignin and cellulose in tobacco leaves, polyphenol contents in different treatments generally rise during flue-curing, while they fall slightly in Stages 3 to 5. The reason for this that this stage is the sensitive period of browning reaction and polyphenol substances are easily oxidised to quinones [32].

During flue-curing, much of the starch is degraded in flue-curing Stages 1 to 3 and 5 and 6: the degradation of starches mainly results from combined action of amylase and amylophosphorylase. During flue-curing, starches are transferred into reducing sugar in the yellowing stage and early stem-drying stage and activities of amylase and amylophosphorylase show two peaks in middle yellowing stage and early leaf-drying stage. At the two peaks, starches are largely degraded [33, 34].

### Chemical quality of tobacco leaves at different ages after flue-curing

130 DAT, 123 DAT, and 116 DAT were roughly ranked from large to small according to the content of reducing sugar of tobacco leaves after flue-curing in the two years, while 116 DAT, 123 DAT, and 130 DAT were ranked from large to small in accordance with content of total nitrogen. In comparison with 116 DAT and 123 DAT, 130 DAT has an older leaf age, so its maturity is higher. With increasing maturity, the content of reducing sugar increases, while content of total nitrogen decreases [35]. With the increase of maturity, tobacco leaves gradually change from C-metabolism and accumulation to N-metabolism, so sugar contents in flue-cured tobacco leaves reduce, while total nitrogen and nicotine rise [36].

Starch metabolism, as the primary metabolism in tobacco leaves, affects the internal quality of tobacco leaves. Starch contents in flue-cured tobacco leaves in different treatments rise with increasing leaf age, because starch contents in tobacco leaves gradually increase with greater maturity. The maturation stage of flue-cured tobacco is a period of rapid synthesis and accumulation of starches in tobacco leaves, and accumulation of starches in tobacco leaves with long leaf age is larger than that at short leaf age. Starch in tobacco leaves peaks at physiological maturity stage and then begins to decrease [37, 38].

Polyphenol, as an important index of measuring quality of tobacco, influences colour and lustre as well as aroma and taste of tobacco leaves. Phenylalanine ammonialyase is the key enzyme used to catalyse the synthesis of polyphenols, while polyphenol oxidase is the key enzyme used to catalyse the degradation of polyphenols. The activities of such two enzymes during flue-curing determine polyphenol contents [39]. Polyphenol contents in tobacco leaves rise with maturity and polyphenol accumulation begins to decrease when reaching physiological maturity [40]. Polyphenol content in flue-cured tobacco leaves at 123 DAT is higher than those at 116 DAT and 130 DAT, indicating that 123 DAT is moderately mature and a lot of polyphenols are accumulated in fresh tobacco leaves. Moreover, phenylalanine ammonialyase of tobacco leaves with a moderate maturity during flue-curing has a high activity and is not easily oxidised into quinone by polyphenol oxidase [41].

### Economic traits of upper tobacco leaves at different ages after flue-curing

Mature tobacco leaves at a proper age have higher quality and economic value. If the leaf is too young, then such short leaves are not matured adequately and their weights are insufficient after picking, which reduces yield and causes economic loss. If a leaf is too long, vegetables such as lettuce, escarole, spinach, Swiss chard are difficult to store and process [13]. In the experiment, the proportion of superior tobacco, average price, yield and output value of upper tobacco leaves in 123 DAT are superior to those in the other two treatments. In comparison with specimens at 123 DAT, 116 DAT shows a shorter leaf age and lower maturity while 130 DAT demonstrates longer leaf age and over-mature tobacco leaves. In the three treatments, the yield of upper leaves at 116 DAT is lowest, because the dry weight of crops is highly correlated with leaf area and the increase of leaf area can increase the dry weight of crops to some extent [42]. Leaf age is an important factor in controlling leaf area index to improve yield [43]. At 116 DAT, the tobacco leaves are young and smaller in length and width, so that less dry matter is accumulated on a small area of leaf.

Previous studies found that tobacco leaves with a high maturity have increasing average price and yield, are golden yellow and contain sufficient oil after flue-curing [44]. In the research, yield, output value, and average price of upper tobacco leaves at 123 DAT were superior to those in the other two treatments, demonstrating that tobacco leaves at 123 DAT were harvested at an appropriate maturity.

## Conclusion

Age of tobacco leaves lays the foundation of leaf structure and chemical compositions and harvesting at an optimal leaf age is an important precondition to ensure quality of tobacco leaves during flue-curing. This study compared physiological and biochemical metabolism of tobacco leaves in treatments of different leaf ages during flue-curing and found that tissue cell gap increased with the increase of leaf age, the thicknesses of upper and lower epidermis as well as palisade and spongy tissues show a W-shaped trend during flue-curing. The older the leaf, the faster the thickness of its structure decreases. With the advance of flue-curing, starch, chlorophyll, carotenoid, and water contents in tobacco leaves at different ages decrease, while polyphenol and MDA contents increase. Furthermore, the older the leaf, the faster the chlorophyll, carotenoid, and water contents reduce during flue-curing, while the faster the polyphenol and MDA contents increase. With the increase of leaf age, contents of total sugar, reducing sugar, and starch in flue-cured tobacco leaves rise, while contents of total nitrogen and nicotine reduce. Flue-cured tobacco leaves at 123 DAT show good economic traits and the highest sensory evaluation score.

The research demonstrates that upper tobacco leaves at 123 DAT were harvested at a leaf age with an appropriate maturity and have an optimal chemical composition after flue-curing. Moreover, their economic value is maximised and the availability of tobacco leaves is improved. For upper tobacco leaves that are too young, pigments and starches degrade slowly during flue-curing and contents of total nitrogen and nicotine are high in such flue-cured tobacco leaves. While flue-curing tobacco leaves at too great an age, water is fast lost and pigments are quickly degraded, so polyphenol and MDA contents in flue-cured tobacco leaves increase. To sum up, 123 DAT can be selected as the quantitative standard for properly harvesting mature tobacco leaves in production.

## Methods

### Experimental location and materials

This study was conducted at Yiliang, Kunming, Yunnan, China (24°91′N 103°14′W). The soil was a sandy red soil (silty loam), which is the dominant soil type for flue-tobacco production in Yunnan. This research site has an altitude of 1539 m, with annual average temperature of 16.3 °C, an annual average total precipitation of 912 mm and 2177 h of annual average sunshine. Details of soil nutrient levels in the research site are provided in Table 6 and a summary of the mean monthly temperatures and total monthly precipitation is provided in Table 7.

**Table 6:** Contents of soil nutrients in Yiliang tobacco-growing area

Soil Type	pH	Organic matter (g·kg^−1^)	Total nitrogen (g·kg^−1^)	Total phosphorus (g·kg^− 1^)	Total potassium (g·kg^− 1^)	Soil available nitrogen (mg·kg^− 1^)	Soil available phosphorus (mg·kg^− 1^)	Soil available potassium (mg·kg^− 1^)
Red soil	6.42	23.96	0.24	0.18	1.92	119.37	29.53	168.74

**Table 7:** The average monthly air temperature and total monthly precipitation (2016 to 2017)

Year	Precipitation						
	April	May	June	July	August	September	Growing season
—————————————mm—————————————							
2016	14.4	58.5	57.6	47.7	136.6	115.2	430
2017	40.5	21.8	178.4	318.4	145.5	125.4	830
————————————Temperature, °C ——————————							
2016	20.2	22.2	22.7	22.7	22.5	20.0	130.3
2107	18.3	21.2	23.1	21.3	22.5	21.8	128.2

### Requirement for field management

Tobacco varieties K326 were provided by Zhongyan Tobacco Seed Co., Ltd., China. For the tobacco plants assessed in the experiment, young seedlings growing under film were transplanted to the field on 15 April 2016 and 18 April 2017, respectively. Pure nitrogen at 90 kg/ha was applied during transplanting and fertiliser at N: P_2_O_5_: K_2_O (1:1:2.5) was applied. More fertiliser from the bottom of the ponds was applied and 50% of the total amount thereof was used as base fertiliser, while 25% was used as seedling promoting fertiliser, and the other 15% was additional fertiliser. All fertiliser was applied 25 d after transplantation. When the tobacco plants grew to 23 to 25 leaves, they were topped: at time of topping (removal of flowers from the top of the plant), two or three useless basal leaves were removed and 20 effective leaves per plant were reserved. After topping on 20 July in 2016 and 2017, chemical suckercides “Maleic hydrazide” (the active ingredient: N-sec-Butyl-4-(2-methyl-2-propanyl)-2,6-dinitroanilin) was applied to suppress buds, so that there were no tobacco flowers on the top and branches on the middle part. Other cultivation measures were applied according to the technical requirements of high-quality tobacco production in Kunming city, Yunnan Province, China.

### Experimental design

**Fig. 12:**
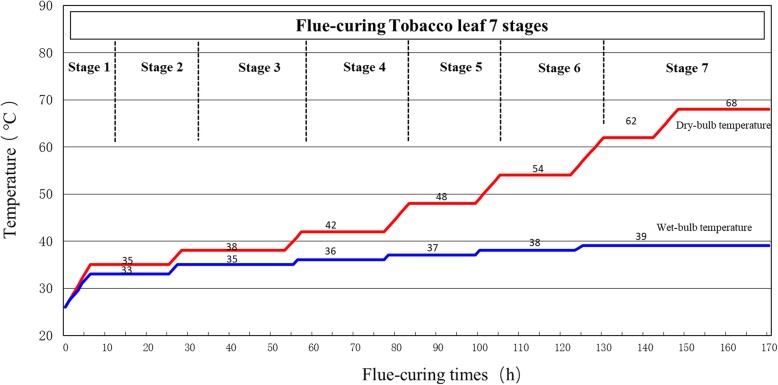
Dry- bulb and wet-bulb temperature in flue-curing stages

The experiment was designed with three different treatments (Table 8), 116 DAT, 123 DAT, and 130 DAT. Different treatments at each time of harvest included approximately 1000 pieces tobacco leaves. The samples of tobacco leaves at different ages were flue-cured with the same flue-curing process. The samples were piecewise collected in seven stages (Fig. 12), i.e. Stage 1 (before flue-curing), Stage 2 (dry-bulb temperature of 35 °C), Stage 3 (dry-bulb temperature of 38 °C), Stage 4 (dry-bulb temperature of 42 °C), Stage 5 (dry-bulb temperature of 48 °C), Stage 6 (dry-bulb temperature of 54 °C), and Stage 7 (after flue-curing).

**Table 8:** Experiment design and experiment code

Year	Transplanting date	Harvested date for upper leaves	Treatment code
2016	15 April	9 August	116 DAT (Days after transplanting)
		16 August	123 DAT (Days after transplanting)
		23 August	130 DAT (Days after transplanting)
2017	18 April	12 August	116 DAT (Days after transplanting)
		19 August	123 DAT (Days after transplanting)
		26 August	130 DAT (Days after transplanting)

### Determination items and methods

#### Structure analysis

Different treatments used nine fresh tobacco leaves and we measured leaf length and leaf width, and the sample leaves were fixed with FAA (50% alcohol) fixative, and specimens were quickly embedded in paraffin and sliced, observed under Motic optical microscope, and statistically analysed for palisade tissue cell gap. The tobacco samples obtained in the seven flue-curing stages were sampled at a position 10 mm from the chief vein in the middle of a leaf by using a hole punch with the diameter of 10 mm and the sampled leaves were placed into formalin–aceto–alcohol (FAA) stationary liquid. A section was made by using paraffin. The section with thickness of 10 μm was dyed with hematoxylin and then thicknesses of upper and lower epidermis as well as palisade and spongy tissues were measured by using a Motic optical microscope. Based on this, structural differences in tobacco leaves at different ages during flue-curing were compared.

#### Analysis of physiological indices

Contents of chlorophyll, carotenoid, water, starch, polyphenol, and MDA of the tobacco samples in the seven flue-curing stages were determined. Chlorophyll and carotenoid were determined by using spectrophotometry, while starch was determined by utilising a continuous flow method [45]. Moreover, high-performance liquid chromatography was used to determine polyphenol and MDA contents. All chemical composition determination methods are determined according to the Chinese tobacco industry standard method, and the measurement results are recognised by the tobacco industry.

#### Economic traits

We had the tobacco samples rated in accordance with National Standard (GB2635–92) and recorded the grading results. According to national purchasing data and supervision and inspection data of grade quality during industry-commerce handover in that year, the average price and proportion of superior tobacco could be estimated. Moreover, based on weight of the tobacco samples, yield, and output value of tobacco leaves were calculated.

#### Analysis of chemical compositions

Contents of total sugar, reducing sugar, starch, and protein were determined by using the Pulse-3000 continuous flow analyser, and total nitrogen was determined by utilising the perchloric-sulphuric acid digestion method. Furthermore, an ultraviolet (UV) spectrophotometric method was applied to determine nicotine contents.

### Statistical analysis

Data analysis was conducted using the General Linear Model (GLM) procedure available in the SAS 9.3 computer package (SAS Institute Inc., Cary, NC). The results indicate that there were significant treatment effects if the probability *P* was smaller than 0.05 for a greater *F*-statistic. Tukey’s honest significant difference (HSD) test was carried out for separation of the means at the 95% confidence level. Sigma Plot 12.3 (Systat Software Inc., Chicago, IL, USA) was used to produce all associated output plots.

## Data Availability

All data generated or analysed during this study are included in this published article [and its supplementary information files].

## Abbreviations

DAT: Days after transplanting
MDA: Malondialdehyde
